# Marine particle size-fractionation indicates organic matter is processed by differing microbial communities on depth-specific particles

**DOI:** 10.1093/ismeco/ycae090

**Published:** 2024-07-12

**Authors:** Jacqueline Comstock, Lillian C Henderson, Hilary G Close, Shuting Liu, Kevin Vergin, Alexandra Z Worden, Fabian Wittmers, Elisa Halewood, Stephen Giovannoni, Craig A Carlson

**Affiliations:** Department of Ecology, Evolution and Marine Biology, Marine Science Institute, University of California Santa Barbara, Santa Barbara, CA 93106, United States; Department of Ocean Sciences, Rosenstiel School of Marine, Atmospheric, and Earth Science, University of Miami, Miami, FL 33149, United States; Department of Ocean Sciences, Rosenstiel School of Marine, Atmospheric, and Earth Science, University of Miami, Miami, FL 33149, United States; Department of Ecology, Evolution and Marine Biology, Marine Science Institute, University of California Santa Barbara, Santa Barbara, CA 93106, United States; Department of Environmental and Sustainability Sciences, Kean University, Union, NJ 07083, United States; Department of Microbiology, Oregon State University, Corvallis, OR 97331, United States; Josephine Bay Paul Center for Comparative Molecular Biology and Evolution, Marine Biological Laboratory, Woods Hole, MA 02543, United States; Faculty of Mathematics and Natural Sciences, Christian-Albrecht University of Kiel, SH, 24118 Kiel, Germany; Josephine Bay Paul Center for Comparative Molecular Biology and Evolution, Marine Biological Laboratory, Woods Hole, MA 02543, United States; Faculty of Mathematics and Natural Sciences, Christian-Albrecht University of Kiel, SH, 24118 Kiel, Germany; Department of Ecology, Evolution and Marine Biology, Marine Science Institute, University of California Santa Barbara, Santa Barbara, CA 93106, United States; Department of Microbiology, Oregon State University, Corvallis, OR 97331, United States; Department of Ecology, Evolution and Marine Biology, Marine Science Institute, University of California Santa Barbara, Santa Barbara, CA 93106, United States

**Keywords:** marine microbiology, biological oceanography, biological carbon pump, 16S amplicon sequencing, particle-associated microbes, Bermuda Atlantic Time-series Study, marine snow, particulate organic matter

## Abstract

Passive sinking flux of particulate organic matter in the ocean plays a central role in the biological carbon pump and carbon export to the ocean’s interior. Particle-associated microbes colonize particulate organic matter, producing “hotspots” of microbial activity. We evaluated variation in particle-associated microbial communities to 500 m depth across four different particle size fractions (0.2–1.2, 1.2–5, 5–20, >20 μm) collected using *in situ* pumps at the Bermuda Atlantic Time-series Study site. *In situ* pump collections capture both sinking and suspended particles, complementing previous studies using sediment or gel traps, which capture only sinking particles. Additionally, the diagenetic state of size-fractionated particles was examined using isotopic signatures alongside microbial analysis. Our findings emphasize that different particle sizes contain distinctive microbial communities, and each size category experiences a similar degree of change in communities over depth, contradicting previous findings. The robust patterns observed in this study suggest that particle residence times may be long relative to microbial succession rates, indicating that many of the particles collected in this study may be slow sinking or neutrally buoyant. Alternatively, rapid community succession on sinking particles could explain the change between depths. Complementary isotopic analysis of particles revealed significant differences in composition between particles of different sizes and depths, indicative of organic particle transformation by microbial hydrolysis and metazoan grazing. Our results couple observed patterns in microbial communities with the diagenetic state of associated organic matter and highlight unique successional patterns in varying particle sizes across depth.

## Introduction

Sinking particulate organic matter (POM) plays a central role in the transport and sequestration of carbon in the ocean, acting as the dominant pathway in the biological carbon pump [1]. Organic particles, comprised of living autotrophic and heterotrophic microbes, cellular detritus, zooplankton-derived carcasses, and fecal matter, can be ballasted by minerals of biogenic and lithogenic origin [2, 3] and account for ~80% of the organic carbon exported annually from the base of the euphotic zone. Physical mixing of suspended organic matter and vertical transport by migrating zooplankton account for the other 20% [4, 5]. Recent studies have shown that the export efficiency of particulate organic carbon (POC) from the surface to depth is spatially variable [6–10]. Factors regulating this spatial heterogeneity remain subject to debate.

Export of POC from the euphotic into the ocean interior is the net outcome of two types of competing processes. Production of POC partially determines particle size, extent of aggregation, and sinking rates and is offset by biological processes that intercept, solubilize, and remineralize sinking organic matter [11, 12]. During transport, particles are transformed by numerous processes, such as direct remineralization by bacterioplankton [13] or zooplankton [14]. Additionally, zooplankton can fragment aggregates [15–17], and POM can be solubilized to dissolved organic matter (DOM) via hydrolytic enzymes produced by particle-associated (PA) prokaryotes [18–21]. These processes result in the attenuation of POM flux by ~85% within the mesopelagic zone (200–1000 m) [11].

Marine POM produced by the aggregation of various types of organic material is colonized by bacteria and archaea that are distinct from the free-living (FL) community. Particles are “hotspots” of microbial activity in the water column [22–24], although the biogeochemical consequences of PA taxa are still poorly understood. PA microbial communities have higher cell densities, growth rates, extracellular enzymatic activities, biogeochemical transformation rates, metabolic diversity, and overall microbial activity per unit volume than surrounding FL communities [18, 20, 25]. These communities degrade POM by producing extracellular enzymes that hydrolyze POM and DOM to lower-molecular-weight compounds prior to cellular uptake [18, 21, 26]. POM and DOM are compositionally distinct pools of organic matter; recently-produced organic particles are less diagenetically altered than surface DOM [27–29]. Therefore, marine POM provides unique microenvironments, leading to microbial communities that are distinct in composition from the surrounding FL communities [22]. The quality and quantity of organic particles are highly variable due to nutrient availability, seasonality, geography, and the distributions of organisms in the water column that produce, consume, and transform POM [2, 7, 30, 31]. Thus, PA communities are highly diverse and may be substantially influenced by several factors, including particle types and quality [32–36], depths, and seasonality [37–40].

This study targeted a greater depth resolution of size-fractionated microbial communities than any previous study we are aware of. The improved vertical resolution allowed us to observe successional patterns in particle communities through depth and directly compare transformations in PA microbes alongside shifts in FL communities. Additionally, we generated both 16S rRNA gene metabarcoding data and complementary isotopic ratio mass spectroscopy data from size-fractionated POM, tying together chemical and microbial characterization. Together, these two data types inform how the diagenetic state of the POM varies along with changes in microbial community structure. The study was conducted in the vicinity of the Bermuda Atlantic Time-series Study (BATS), a site with rich contextual data for primary productivity, vertical fluxes and biogeochemical variability [41, 42], DOM dynamics [28, 43–45], microbial processes [46–48], microbial plankton diversity [49–55], and sinking particles [56]. We deployed *in situ* pumps (McLane large volume water transfer systems) equipped with filters of successively smaller pore sizes (20, 5, 1.2, 0.2 μm) to capture particle size fractions. This approach captures sinking particles as well as suspended particles, which differs from gel and sediment traps that only collect the sinking fraction [57, 58]. Additionally, *in situ* pumps filter hundreds of liters, a far greater volume than the most popular method for PA community analysis, in which water collected from Niskin bottles is sequentially filtered [59]. With these data sets, we explored changes in PA communities and transformations of organic matter in different size fractions and across depths. We used this data to ask: (i) to what extent do PA communities change with depth, (ii) do PA communities within the mesopelagic become more compositionally distinct from FL communities as the organic particles are diagenetically transformed, and (iii) can we resolve certain taxa shifting between a FL and PA lifestyle across depth. Our study constrains drivers of particle transformation and connects observed biogeochemical transformations to associated microbial taxa, emphasizing the various roles microbes play in marine biogeochemical cycling.

## Materials and methods

### *In situ* pump and hydrographic sampling

Size-fractionated samples were collected by McLane *in situ* pumps (McLane Research Laboratories, Inc.) outfitted with 4 L min^−1^ pump heads and dual filter heads, each containing four-tiered 142 mm filter holders. One filter head collected microbial DNA fractions, and the other collected size-fractionated particles for organic chemical characterization. Fifty-two samples were collected between the surface and 500 m over four cruises conducted in the vicinity of BATS in July 2018, July 2019, August 2021, and November 2021. Three to four *in situ* pumps targeting different depths were deployed on a hydrowire simultaneously, filtering between 47 and 367 L of seawater through each pump head over time intervals of up to four hours. DNA was collected via sequential filtration through a 20 μm Nitex screen with a 150 μm Nitex backing filter, a 5 μm polycarbonate isopore (TMTP) filter, a 1.2 μm polyethersulfone (PES, Pall Corporation) filter, and a 0.2 μm PES filter. Samples for organic characterization were collected sequentially on a 20 μm acid and methanol-cleaned Nitex mesh, a 6 μm acid and methanol-cleaned Nitex mesh, a 1.2 μm GF/C glass microfiber filter (double layer, precombusted at 450°C), and a 0.3 μm GF-75 glass microfiber filter (double layer, precombusted at 450°C).

### Size fractionated DNA preservation and extraction

Immediately after recovery, pump heads were drained of standing volume by a gentle vacuum. DNA filters were transferred from the pumps to polyethylene bags, heat sealed, and stored at −80°C until lysis and extraction. For cell lysis, 6 ml of sucrose lysis buffer (40 mmol L^−1^ EDTA, 50 mmol L^−1^ Tris HCl, 750 mmol L^−1^ sucrose, 400 mmol L^−1^ NaCl, pH adjusted to 8.0), 600 μl of sodium dodecyl sulfate (10% w/v), and 60 μl of 20 mg ml^−1^ proteinase K were added to each polyethylene bag. Bags were then resealed and incubated at 37°C for 30 min and then at 55°C for 30 min. A 1 ml aliquot was transferred to 2 ml microcentrifuge tubes for DNA extraction. DNA was extracted following the phenol-chloroform protocol of Giovannoni *et al.* [55].

### Amplicon library sequencing and bioinformatics

Amplification of the V4 region of the 16S rRNA gene was performed using the 515F-Y (5′-GTGYCAGCMGCCGCGGTAA-3′) and 806RB (5′-GGACTACNVGGGTWTCTAAT-3′) primers with custom adapters [60, 61]. PCR-grade water process blanks and mock communities (BEI Resources mock communities HM-782D and HM-783D and a custom community from the Santa Barbara Channel [62]) were included with each 96-well plate of samples. Amplicons were cleaned and normalized using SequalPrep plates (Invitrogen), pooled at equal volumes, concentrated using Amicon Ultra 0.5 ml centrifugal tubes (Millipore), gel extracted using the QIAquick Gel Extraction Kit (Qiagen), and sequenced on an Illumina MiSeq using PE250 chemistry at the University of California, Davis DNA Technologies Core.

Fastq files were processed using dada2 (version 1.16) in R [63]. Amplicon sequence variants (ASVs) were given a taxonomic assignment using the SILVA database (version 138.1 with species) [64]. *Prochlorococcus* taxonomy was manually refined, and sequences were assigned to ecotypes using phylogenetic approaches based on sequence alignment against representative *Prochlorococcus* 16S rRNA gene sequences. Samples were limited to 8000 reads. Samples with fewer than 8000 reads were removed from further analysis (25 out of 468 samples). Mock communities and negative controls were compared to confirm consistency in amplification and lack of contamination between PCR plates and then removed from further analysis. ASV relative abundances were pretreated using an angular transformation to normalize the dataset, and singletons were removed for all multivariate analyses.

### Particle characterization

Organic particles were chemically characterized as described previously [65, 66]. Briefly, particles were collected sequentially on 20, 6, 1.2, and 0.3 μm filters. Isotope samples were collected on combusted glass fiber filters with nominal pore sizes to avoid the organic matrix of polymer filters, which render bulk C&N analysis impossible. DNA samples were collected on polycarbonate and polyethersulfone flat filters with exact pore size cutoffs. Note that this may have led to the collection of slightly different particles on differing filter types. Particles in the >20 μm and 6–20 μm fractions were washed off Nitex filters with 0.2 μm filtered seawater and collected onto 0.7 μm GF/F glass fiber filters. Filters were examined under magnification to identify and remove metazoan swimmers captured during sampling. Filters were then freeze-dried and quantitatively split by weight, as in Graff *et al.* [67]. For bulk nitrogen and amino acid analysis, freeze-dried filters were processed directly. For bulk analysis of organic carbon, filter subsamples were acidified via direct application of saturated sulfurous acid and dried at 60°C overnight. Bulk POM nitrogen concentrations and stable isotope ratios were measured via an elemental analyzer coupled to an isotope ratio mass spectrometer (Flask EA coupled to MAT 253-IRMS via Conflo IV, Thermo Scientific). Standards of known nitrogen isotope composition (Arndt Schimmelmann, Indiana University) were used to calibrate instrument response and precision; analytical uncertainty in bulk nitrogen isotope composition was ±0.2‰.

For amino acid compound-specific isotope analysis, splits from 0.3, 1.2, and 20 μm filters were freeze-dried, hydrolyzed, purified, derivatized, and analyzed via gas chromatography coupled with isotope ratio mass spectrometry (GC-IRMS) as previously described [65, 68]. POM captured on 6 μm filters was below detection and thus excluded for isotopic analysis. Norleucine and aminoadipic acid co-injection standards and a standard mixture of 14 amino acids with known δ^15^N values were run alongside samples to correct for instrument drift, potential peak area relationships, and possible isotope effects associated with derivatization.

Trophic position (TP) of POM was estimated based on measured δ^15^N values of glutamic acid (Glu) and phenylalanine (Phe), as in Chikaraishi *et al.* [69]:


(1) \begin{equation*} \mathrm{TP}=\left({\mathrm{\delta}}^{15}\mathrm{N_{Glu}}-{\mathrm{\delta}}^{15}\mathrm{N_{Phe}}-3.4\right)/7.6+1 \end{equation*}

TP uncertainty was determined, as in Jarman *et al.* [70]. Due to the large amount of material and processing time required for amino acid δ^15^N measurements, amino acids were analyzed and TP was calculated for only a subset of samples.

## Results

### Hydrography

The depth of the surface mixed layer varied between and within cruises, from 9 m during stratified summer periods to 81 m in November 2021. Average surface temperatures ranged from 28.9°C in August 2021 with a mixed layer of ~9 m to 24.6°C in November 2021 with a mixed layer of ~70 m. The deep chlorophyll maximum (DCM) varied between 69 and 134 m. The depth of the DCM ranged from 69 to 88 m in July 2018, 81 to 100 m in July 2019, 101 to 134 m in August 2021, and 77 to 93 m in November 2021. Microbial communities binned as being associated with the DCM fell within these depth ranges of ±10 m for each cruise. Profile data for temperature, chlorophyll, bacterial abundance, nitrate + nitrite, POC, and DOC during all sampled time periods can be found in Fig. S1 and broadly match values and trends previously reported at BATS for the summer and autumn seasons [41, 42].

### Chemical characterization of organic particles

**Figure 1:**
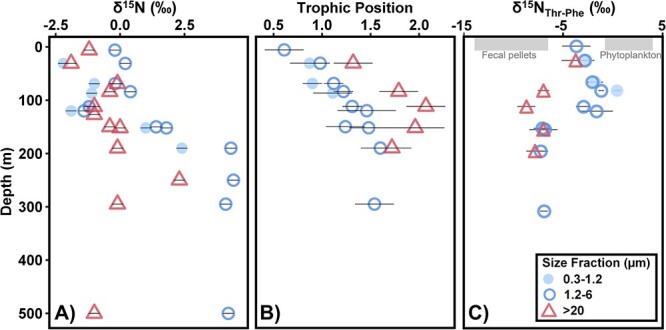
(A) Separation of bulk δ^15^N values between fractions was observed below the DCM. (B) TP of particles was highest in the >20 μm fraction across all depths. (C) δ^15^N values threonine (Thr) normalized to δ^15^N values of phenylalanine (Phe) for POM size fractions show differing composition between size fractions. For comparison, the range of values found in phytoplankton and zooplankton fecal pellet end-members characterized by Doherty *et al.* (2021) are indicated by gray bars at the top. Additional comparison to end-members can be found in Fig. S7. Error bars indicate propagated analytical uncertainty. Samples were taken from July 2018.

Bulk δ^15^N values and TP were measured from the same pump casts that DNA was collected from in July 2018. Bulk δ^15^N values were used to estimate organic matter degradation. Higher values indicated more bioavailable organic matter consumption due to preferential biological utilization of the lighter isotope (^14^N) leaving behind a more ^15^N-enriched substrate [71]. Bulk δ^15^N values ranged from −2‰ to 4‰, varying between size fractions and depth (Fig. 1A). Values between fractions in the upper euphotic (UE) were more similar than those observed between fractions below the DCM. Bulk δ^15^N values in the <6 μm fractions were lowest in the surface 100 m, then increased sharply from −2‰ to 4‰ between 120 and 190 m (Fig. 1A). There were not sufficient concentrations of POM in the 6–20 μm size fraction to accurately measure bulk or compound-specific δ^15^N values. No significant increase in δ^15^N values was observed over depth in the >20 μm fraction. Instead, a δ^15^N value ≤0‰ was observed for all mesopelagic depths except 250 m, which had a δ^15^N value of 2.3‰. We use the bulk δ^15^N values of size-fractionated POM from July 2018 to represent all other stratified periods sampled in this study because of the similarity in hydrography, nutrient fields, euphotic zone depths, and location and intensity of the DCM. Bulk stable isotope analyses performed across these sampling periods demonstrate similar patterns, suggesting these observed trends are relevant to all sampled time periods [72].

TP of particles was calculated from measured δ^15^N values of individual amino acids for the 0.3–1.2, 1.2–6, and >20 μm fractions from several depths in July 2018 (Fig. 1B). The TP determines the extent to which particles include organic matter produced through catabolic processes [73–75], with a higher TP indicating greater contributions from heterotrophic microbial biomass or solid waste from animals. The lowest TPs (<1.0) were observed at the surface and increased with depth. TP was only calculated from three samples within the 0.3–1.2 μm fraction; however, at each depth, this fraction aligned with values observed in the 1.2–6 μm fractions and displayed a significantly lower TP than the >20 μm fraction. The highest values were observed at 190 m (1.6 ± 0.2) for the 1.2–6 μm fraction and at 112 m (2.1 ± 0.2) for the >20 μm fraction. At all depths sampled, the >20 μm fraction displayed the highest calculated TP; along with relatively low δ^15^N values of threonine (measured through compound-specific isotope analysis of amino acids (CSIA), Fig. 1C), this suggests greater metazoan influence on the processing of the larger particles [66].

### Overall trends in FL and PA bacterioplankton communities

**Figure 2:**
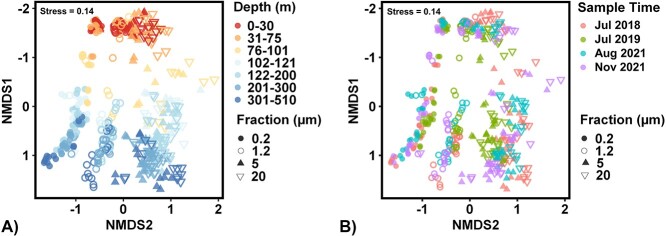
NMDS ordinations show strong differences in microbial community composition are observed between fraction and depth. NMDS ordinations use bray-Curtis dissimilarities from 16S rRNA gene amplicon community composition, with *Cyanobacteria* and plastid sequences excluded. Shapes denote size fraction. Colors denote (A) depth within the water column and (B) time of collection.

Overall, 432 environmental samples were successfully sequenced and contain 10 907 non-singleton ASVs after rarefaction. Eukaryote and plastid sequences were removed prior to analysis to focus on prokaryote diversity. Communities within the 0.2–1.2 fraction were considered FL, while communities within the 5–20 and >20 μm fractions were considered PA, consistent with previous studies of size-fractionated microbial communities [32, 40]. Cyanobacterial contributions were most pronounced in the 0.2–1.2 μm (17.6 ± 9.5% of total 16S community in the UE and 26.9 ± 10.1% in the DCM) and 1.2–5 μm (29.1 ± 13.1% in the UE and 35.7 ± 10.2% in the DCM) fractions in the UE and DCM but were present in all size fractions at all depths to 500 m (Fig. S4, see Supplementary Material). Nonmetric multidimensional scaling (NMDS) ordination demonstrated a similar pattern with and without the inclusion of *Cyanobacteria* (Figs 2 and S2).

Unless otherwise specified, for subsequent analyses, cyanobacterial taxa were removed to directly compare FL and PA heterotrophic and chemoautotrophic communities. Permutational multivariate analyses of variance (PERMANOVA) was used to identify potential environmental drivers of prokaryotic community variability. NMDS ordinations revealed distinct clustering by size fraction and sample depth (Fig. 2A). Size fraction (*R*^2^ = 0.15, *P* < .001) and depth (*R*^2^ = 0.12, *P* < .001) were the strongest drivers of community composition in this dataset. Sampling date explained the lowest proportion of variance (*R*^2^ = 0.06, *P* < .001). When cyanobacterial taxa are considered, the environmental factors that affect community variations remain the same. The size of the particle, the depth of the sample, and the date of sampling all have a significant impact on the photoautotrophic, heterotrophic, and chemoautotrophic communities. The statistical analysis showed that the size fraction, depth, and sampling date all have a significant relationship with community variability (*R*^2^ = 0.15, *P* < .001; *R*^2^ = 0.15, *P* < .001; *R*^2^ = 0.06, *P* < .001, respectively).

### Community variation within the upper euphotic, DCM, and twilight zone depths

Sample depths were grouped into three categorical bins to investigate broad patterns across gradients of light, organic, and inorganic nutrient fields within the surface 500 m. We defined the UE zone as depths between the surface and 10 m shallower than the maximal DCM fluorescence. Samples collected within ±10 m of maximal fluorescence for each cast were binned as “DCM” samples, and samples collected >10 m deeper in the DCM were binned as the “twilight zone.” Within all sample depth bins, PERMANOVA analysis indicated that the size fraction of the sample was a significant driver of community variation (PERMANOVA *R*^2^ = 0.32–0.33, *P* < .001), suggesting that PA and FL community structure differed across all depths.

**Figure 3:**
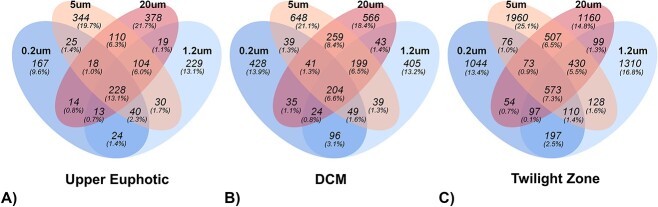
A lower percentage of ASVs overlap between fractions below the UE. Venn diagrams show unique and overlapping ASVs between the 0.2–1.2, 1.2–5, 5–20, and >20 μm size fractions in (A) the UE, (B) the DCM, and (C) the twilight zone.

A multivariate analog of Levine’s test for homogeneity of variances was used to assess the similarity of communities within each depth, followed by Tukey *post-hoc* analyses to assess significance. Communities within the UE bin had smaller average distances from the calculated mean than communities at depths in the twilight zone (Fig. S6), implying that size-fractionated communities in the UE were more similar to each other than communities at greater depths. Within the UE, 13.1% of ASVs were present in all size fractions (Fig. 3). However, within the DCM and twilight zone, only 6.6%–7.3% of ASVs were shared between all fractions, supporting the finding that there was less overlap in FL and PA taxa below the UE.

### Community variation within size fractions

**Figure 4:**
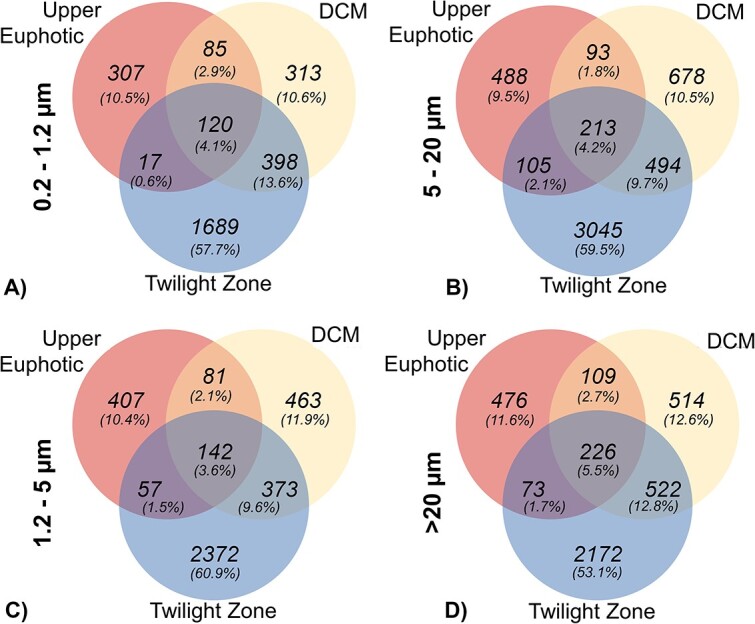
There is a low percentage of overlap between ASVs in the UE and the DCM or twilight zone. Venn diagrams show unique and overlapping amplicon sequence variants (ASVs) between depth bins associated with the UE, the DCM, and the twilight zone in (A) the 0.2–1.2 μm size fraction, (B) the 1.2–5 μm fraction, (C) the 5–20 μm fraction, and (D) the >20 μm fraction.

PERMANOVAs confirmed that depth was a strong driver of community structure variability within each size fraction (*R*^2^ = 0.38–0.43, *P* < .001). The degree of variance between communities in the 0.2–1.2, 1.2–5, and >20 μm fractions (*R*^2^ = 0.41–0.43, *P* < .001) was similar to that observed over depth and slightly reduced in the 5–20 μm fraction (*R*^2^ = 0.37, *P* < .001). For all size fractions, there was only minor overlap of ASVs (3.6–5.5%) in the UE, DCM, and twilight zone depth bins (Fig. 4), suggesting specialization across depth in all size fractions.

### Alpha diversity

**Figure 5:**
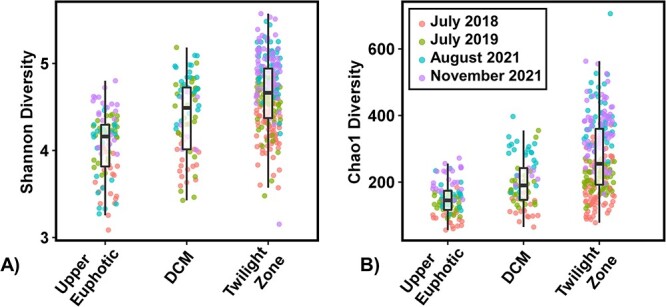
Significant differences in alpha diversity of the prokaryotic community based on 16S rRNA gene amplicons (excluding *cyanobacteria* and plastids) were observed between depth bins and timepoints in all size fractions.

Shannon diversity indices within all size fractions differed significantly over depth (ANOVA *P* < .001) as well as seasonally (ANOVA *P* < .001) (Fig. 5A). Within the UE, there was a weak but significant increase in Shannon diversity with increasing fraction size (*P* = .013). However, no significant differences in Shannon diversity were observed between size fractions within the DCM and twilight zone (ANOVA *P* = .238). Trends in Chao1 were similar to those observed with Shannon diversity (Fig. 5B) and were not affected when *Cyanobacteria* were included (Fig. S3).

### Distributions of cyanobacterial lineages

**Figure 6:**
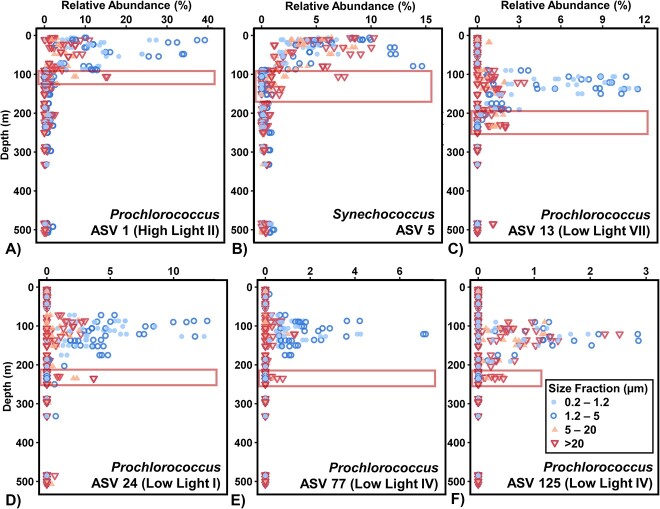
Certain cyanobacterial ASVs demonstrated a higher relative abundance in larger fractions directly below their free-living max depths range. Boxes denote depths where *Cyanobacteria* are found with greater relative abundance in the PA size fractions compared to free-living fractions.

*Cyanobacteria* reached a maximum of 54.5% of amplicons from the prokaryotic community and were predominantly *Prochlorococcus*. In all size fractions, *Cyanobacteria* were higher in relative abundance in the UE and DCM compared to the twilight zone (Fig. S4). The greatest relative abundances of both *Prochlorococcus* and *Synechococcus* were observed in the 0.2–1.2 and 1.2–5 μm fractions, with corresponding ASVs having a slight but significantly greater relative abundance in the 1.2–5 μm fraction (Tukey HSD *P* < .05). *Prochlorococcus* (ASV #1) and *Synechococcus* (ASV #5) had the highest relative abundances in the upper 100 m of the water column. A greater number of less abundant cyanobacterial ASVs had higher relative abundances within and just below the DCM (Fig. 6). While all cyanobacterial ASVs were found in the highest relative abundance in the <5 μm size fractions, many cyanobacterial ASVs increased in relative abundances in the 5–20 and >20 μm fractions at the base of the euphotic zone and the twilight zone at depths several meters below the depth range where their respective FL (<5 μm) maximum occurred.

### Distribution of non-photosynthetic prokaryotes across size-fractions

**Figure 7:**
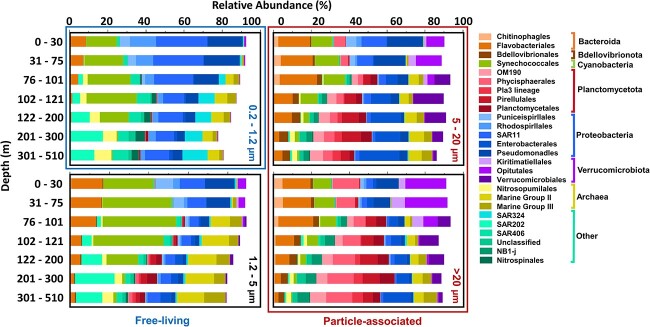
Average relative abundances of microbial taxa (including *Cyanobacteria*) grouped by order across size fractions and depth bins. Orders that comprised <1% of community composition were excluded from visualization.

Microbial communities were analyzed with cyanobacterial ASVs removed to evaluate patterns of heterotrophic and chemoautotrophic prokaryotes. Between size fractions, significant differences were observed in the relative abundance of all major taxonomic groups. The FL 0.2–1.2 μm fraction was significantly (Tukey HSD *P* < .01) enhanced with *Rhodospirillales* (AEGEAN-169, Magnetospiraceae), SAR11 (Clade I, III, and IV), and SAR324 compared to every other size fraction (Figs 7, S4, and S5). *Rhodospirillales* averaged 14.5% of the 0.2–1.2 μm fraction community in the UE, while SAR11 averaged 32.1%. SAR324 was most abundant in the twilight zone FL community, averaging 10.4%. Additionally, the relative contribution of *Flavobacteriales* was significantly lower in the 0.2–1.2 μm fraction relative to all other fractions. The 0.2–1.2 and 1.2–5 μm fractions were enriched with SAR202, SAR406, SAR324, SAR11, *Puniceispirillales*, *Rhodobacterales*, *Rhodospirillales*, *Nitrospinales*, Marine Group II (MG II), Marine Group III (MG III), and *Nitrosopumilales* compared to the 5–20 and > 20 μm fractions, suggesting these taxa are largely FL (Fig. 7). The 1.2–5 μm fraction was enriched with MG II and MG III in the twilight zone with an average of 16.5% and 7.9%, respectively.

The 5–20 and >20 μm fractions were each enhanced in *Gammaproteobacteria* (*Enterobactales*), *Verrucomicrobiota* (*Kiritimatiellales*, *Opitutales*, and *Verrucomicrobiales*), *Planctomycetota* (OM190, *Phycisphaerales*, Pla3, *Pirellulaes*, *Planctomycetales*), and *Bacteroidota* (*Chitinophagales* and *Flavobacteriales*). *Enterobactales* were most abundant in the 5–20 μm fraction in the twilight zone, averaging 14.7% of the non-cyanobacterial community. A member of the genus *Alteromonas*, part of the class Enterobactales, represented the single most abundant ASV found in the PA fractions, reaching up to 29.2% of the non-cyanobacterial PA communities. Verrucomicrobiota were highest in the UE and DCM, averaging 13.8% and 28.5% of the communities in the UE 5–20 μm and >20 μm fractions, and 26.0% and 31.5% in the DCM 5–20 um and >20 μm communities. *Planctomycetota* averaged 26.0% of the 5–20 μm community and 36.4% of the >20 μm community within the twilight zone. Bacteroidota displayed enhanced abundance in all >1.2 μm fractions relative to the FL fraction, averaging 22.9%–25.4% of the >1.2 μm fractions within the UE, where it was most abundant.

### Heterotrophic and chemoautotrophic taxonomic succession over depth

There was strong vertical stratification of non-cyanobacterial prokaryotes across the sampled depth range. While community composition varied across size fractions, each size fraction demonstrated similar vertical stratification in taxa over the upper 500 m. Within the UE of all size fractions, ASVs assigned to members of the orders *Opitutales*, *Flavobacteriales*, *Chitinophagles*, and various *Proteobacteria* (*Pseudomanadles*, *Puniceispirillales*, *Rhodospirllales*, and SAR11) were observed in significantly higher relative abundance than in the twilight zone (Figs 7, S4, and S5). Greater relative abundances for members of SAR202, SAR406, SAR324, *Alteromonas*, *Verrucomicrobiales*, *Nitrospinales*, NB1-j, *Bdellovibrionales*, *Planctomycetota* (OM190, Pla3, *Pirellulales*, and *Planctomycetales*), and *Archaea*, including *Nitrosopumilales*, MG II, and MG III, were observed in the DCM and twilight zones in all size fractions compared to the UE zone.

## Discussion

### Characterization of marine organic particles

The observed increase in particulate organic δ^15^N values of ~4‰ from the surface to 500 m is consistent with previous observations around the global ocean [68, 71, 76, 77]. The shift in the δ^15^N values is accompanied by changes in particle composition. Fresh phytoplankton material dominates surface POM, while particles from the twilight zone are enriched with detritus [78, 79]. The increase in δ^15^N values is attributed to the preferential solubilization of ^14^N-containing bonds during extracellular protein hydrolysis, leaving behind partially degraded particles enriched in ^15^N [71]. This interpretation is consistent with the findings of Hannides *et al.* [68], who reported that the primary driver of ^15^N-enrichment in suspended particles was microbial heterotrophic degradation rather than metazoan grazing or a shift in nitrogen source. This suggests that smaller particles were largely transformed by PA microbial communities, which were dominated by members of *Planctomycetota*, *Verrucomicrobiota*, *Bacteroida*, and *Gammaproteobacteria*.

In contrast to the smaller (<20 μm) fractions, we did not observe significant changes in δ^15^N values over depth in large (>20 μm) particles. δ^15^N values of large, sinking particles have previously been observed to increase only slightly [77] or even decrease [76] with depth, despite rapid attenuation below the euphotic zone. Thus, processes that transform larger particles differ from the microbial processes affecting smaller particles [76]. These findings are consistent with previous reports of small, suspended particles being isotopically distinct from large, sinking particles [80, 81]. Therefore, bulk δ^15^N values alone could not differentiate whether large particles were sinking rapidly through the water column relatively untransformed, or whether the bulk δ^15^N signal did not fully capture biogeochemical transformations in large particles.

To better distinguish drivers of nitrogen isotopic composition and particle degradation within particle size fractions, we used CSIA. The interpretive power of this method derives from differing responses of amino acids (AA) to trophic transfer, whereas during microbial hydrolysis, the enzymatic transformation of amino acids is similar and untargeted [82]. One group of “trophic” AAs (i.e. alanine, aspartic acid, glutamic acid, isoleucine, leucine, proline, and valine) becomes significantly enriched in ^15^N with every trophic transfer. Another group of “source” AAs (i.e. glycine, lysine, methionine, phenylalanine, and serine) experiences little change in ^15^N during trophic transfers, instead retaining their source δ^15^N ratio [69]. TP can be estimated through differences in δ^15^N values between the trophic AA glutamic acid/glutamine (Glx), and the source AA phenylalanine (Phe; Equation 1) [69]. The concept of TP has recently been applied to water column particles, with TPs >1 indicative of contributions from heterotroph biomass or solid waste products, both associated with consumers performing amino acid catabolism [66].

Here, TP increased from near-surface to 500 m in both small and large particles, indicating increases in biomass or waste contribution from heterotrophic organisms, whether microbial or metazoan [73–75]. We observed higher TPs at all depths in the largest (>20 μm) fraction, with the highest TPs found below 100 m (Fig. 1B). Independent of depth, the higher TP of larger particles suggests disproportionate transformation by catabolic processes compared to smaller particles, which primarily showed evidence of hydrolytic transformation. Threonine, one of the amino acids measured with CSIA, can inform metazoan waste contribution to TP, with low δ^15^N values of threonine in large particles indicating that their elevated TP of large particles is due to metazoan waste contribution [66]. The crucial role of mesozooplankton grazers in organic particle packaging, transformation, and carbon flux has been widely acknowledged [83–86]. Through consumption of phytoplankton and detrital particles, zooplankton can produce dense fecal pellets, thereby repackaging small, suspended particles into larger, denser, and potentially faster sinking particles [87]. Disaggregation of these fecal pellets can also influence the TP of smaller size fractions [66, 80]; however, the observed higher TP of the >20 μm size fraction suggests greater metazoan transformation of larger particles. Similar to previous findings, this metazoan alteration is not apparent from bulk δ^15^N values [66].

### Drivers of community differentiation

Differences in community structure and metabolic potential between PA and FL bacterioplankton communities are well-established [22]. Variability in POM source, composition, and micro-environmental conditions relative to the surrounding water column have been reported to correlate with community structure variability [23, 32, 40, 56, 88]. The variability in microbial community structure is linked to the physiological and metabolic differences of PA taxa. These taxa often consist of large cells with high respiration rates [25] and expanded gene repertoires for breaking down polysaccharides [40, 89–92]. Additionally, they have large genome sizes [89]. It is well documented that FL bacterioplankton taxa are highly stratified from the UE zone into the mesopelagic zone in subtropical gyres, presumably resulting from niche partitioning across gradients of energy and available organic and inorganic nutrients [52, 53, 55, 93]. Our data shows that while the composition of PA communities was distinct from the FL communities, there was a similar degree of community structure stratification over depth community shift from the euphotic zone into the twilight zone. This is noteworthy as previous findings have shown less community succession in larger particles [94]. The colonization of organic particles by bacteria and archaea corresponds with changes in nutrient composition and associated POM bioavailability [32–36]. If most particles were rapidly sinking and remained relatively untransformed, we might expect PA communities to be more similar over depth than FL communities. However, all particle-size fractions appeared to undergo significant chemical and microbial compositional transformations through the water column. These transformations are likely driven by compositional changes in the particulate substrate due to processes, including microbial reworking [13, 18, 19] and zooplankton consumption, fragmentation, and repackaging [14, 15, 16, 20]. The succession of PA communities observed over depth indicates that a considerable portion of the particles collected by the *in situ* pumps are non-sinking or slowly sinking particles. These suspended particles are home to taxa that are adapted to living in a particular depth range, across gradients in nutrient fields or particle composition [95, 96]. Alternatively, the rapid turnover of particle microbial communities as they sink could explain our observations.

The increase in alpha diversity in the FL and PA communities below the DCM is consistent with previous observations [36]. POM and DOM in the twilight and mesopelagic zones consist of a wide range of chemically distinct compounds that are more diagenetically altered and less labile compared to organic matter in the UE [28, 97]. This may support increased functional and phylogenetic diversity in prokaryotic communities over depth. For example, the increasing alpha diversity we observe over depth in every size fraction may be indicative of a shift in organic matter composition in which the common easily degradable compounds become scarce, and a variety of organic compounds that require more specialized pathways for degradation accumulate at deeper depths [28, 97]. In addition to mesopelagic communities being more diverse, they also become more different from one another across fractions. This increasing differentiation in communities within the mesopelagic has previously been observed between sinking and suspended particles [34]. Both POM and DOM in the surface ocean originate largely from phytoplankton; however, labile POM and DOM are rapidly consumed, with only a fraction resisting degradation long enough to be exported into the mesopelagic [28, 98]. In addition to DOM becoming more diagenetically altered with depth, larger organic matter fractions have been shown to be less diagenetically altered with depth than DOM [27, 97]. Differences in organic matter composition over depth are also revealed from isotopic signals discussed previously. These differences can be selected for differing communities that consume and transform this organic matter. While both POM and DOM are consumed and transformed by microbes, communities associated with FL and PA lifestyles are significantly different and contain differing metabolic potentials, including more polysaccharide degradation and amino acid transport genes within the PA communities [38, 86]. The resulting recalcitrant material of different size fractions reworked via differing metabolic processes, both microbial and metazoan, may require specialized microbial communities to break down the remaining material, potentially causing increased dissimilarity between FL and PA communities below the DCM (Fig. 2A).

Seasonal variability in FL and PA communities is widely observed at BATS and in the global ocean [37, 52, 53, 56, 99]. However, this dataset only contains samples collected in summer and autumn, so seasonal variability is underrepresented. The magnitude of variability observed between timepoints was less than half that observed between fraction or depth. During stratified periods at BATS, the temporal variability in the bacterial communities between size fractions and over depth observed was minimal. This suggests that it is not a primary driver in community composition in FL and PA communities, as shown in Fig. 2B. The interannual variability between 2018 and 2021 for the stratified periods was relatively similar, which is consistent with stable interannual patterns of size-fractionated prokaryote communities observed in other systems [37].

### Microbial taxa distributions

The PA 5–20 and >20 μm fractions displayed an increased relative abundance of members of *Bacteriodota*, *Proteobacteria*, *Verrucomicrobiota*, *Bdellovibrionota*, and *Planctomycetota*, relative to the FL fractions. Members of these taxa are commonly the most dominant clades found in PA communities [100]. While we cannot determine metabolic potential from amplicon data alone, previous studies suggest that taxa will exploit varying nutritional niches as particle composition becomes altered. For example, while members of *Alphaproteobacteria* can be more efficient in the incorporation of monomers and amino acids, members of *Bacteriodota* (specifically *Flavobacteriaceae*) developed enzymatic repertoires to degrade higher molecular weight compounds [101, 102]. Members of *Gammaproteobacteria* are important degraders of algal polysaccharides, an abundant component of phytoplankton-derived POM [103]. *Planctomycetota* have been predominantly found in larger particle fractions [22, 104] with genomic analyses revealing several members within that clade to be capable of degrading complex organic matter [105].

Members of *Rhodobacteraceae*, also commonly associated with marine particles [100], were found in higher abundance in smaller 0.2–1.2 and 1.2–5 μm fractions, though they were present in larger fractions as well. Several other taxa, including members of *Bacteriodota* (*Flavobacteriaceae*), *Gammaproteobacteria*, *Bdellovibrionia*, and *Planctomycetota*, were found in detectable abundances in all fractions. This could be due to a “stick or swim” lifestyle where some microbes can switch between FL and PA phases of taxa depending on environmental conditions [106]. Alternatively, there could be attachment due to particle stickiness [107], niche partitioning between members of the same taxa [108, 109], or retention of FL taxa on larger filters, and concomitant fragmenting of particle communities during filtration. We considered whether the potential retention of FL taxa with concomitant particle fragmentation would create more similar communities between size fractions, making it more difficult to determine the potential lifestyle of various taxa. Notably, the striking differences in microbial community structure observed in this dataset demonstrate that, despite these caveats, size fractionation is nonetheless effective at separating functional components of microbial communities by size.

A previous study of POM collected via sediment traps at BATS revealed that *Cytophagales*, *Chitinophagales*, and *Cellvibrionales* were indicator taxa of sinking particles [56]. The *in situ* pump collection method used in the present study demonstrated a relatively low abundance of these taxa within both PA and FL communities. Notably, *in situ* pump collections differentiate only coarsely between fast sinking, slow sinking, and suspended particles. Differences in the microbial community structure of sinking and suspended particles in the Scotia Sea have been reported [34]. The observed differences in particle community composition between our study and Cruz *et al.* [56] likely emphasize that a majority of organic particles at BATS are suspended or slowly sinking rather than rapidly sinking like those preferentially collected in sediment traps. However, consistent with Cruz *et al.* [56], we observed *Alteromonas* and *Vibrionales* to be in the greatest relative abundance in the >5 μm fractions, which would include fast-sinking particles caught in sediment traps. These results emphasize the utility of *in situ* pumps in resolving trends in bulk particulate material. Differences in sinking and suspended PA communities have previously been reported, and are associated with varying lifestyles, with sinking particles being associated with R-strategists and suspended particles being associated with K-strategists, a trend driven by the differing quality of organic matter [34].

Notably, these bulk collection methods may obscure potentially differing trends and communities between particles of differing origins. Stephens *et al.* [110] observed differing microbial communities on different sinking particle types in the Pacific Ocean, a nuance that is obscured with bulk measurements. Additionally, Stephens *et al.* observed decreasing alpha diversity on individual particles with increasing depth, the opposite trend observed in this study, where alpha diversity increases below the DCM. These differences may be explained by the different particle types collected, with Stephens et al. collecting sinking particles from traps, whereas we collected bulk material. Sinking particles have been hypothesized to have decreasing alpha diversity with depth, whereas suspended particles have increasing microbial diversity with depth, further supporting our hypothesis that a majority of particles collected are suspended [34].

### *Cyanobacteria* found in particles at depths below their maximum free-living abundance

Large particles can be fragmented via physical shear, zooplankton feeding [15, 17], and enzymatic solubilization by attached microbial communities [18, 111]. While ASVs found in the highest abundance in the PA fractions were also present in the FL fractions, we were not able to resolve the PA taxa transition to the FL fraction for any depths sampled. The FL communities resemble whole seawater communities previously sampled at BATS [52, 53], suggesting that FL microbes are abundant enough to largely obscure PA communities during sequencing of whole seawater. Due to the scarcity of marine particles in the water column, PA bacteria make up only around 1% of the total prokaryotic community [112, 113]. Therefore, while fragmentation of particles may be occurring, these taxa could be vastly outnumbered by ambient FL communities, and therefore the transition to a FL lifestyle cannot be captured effectively by our approach.

*Synechococcus* and *Prochlorococcus* were observed shifting to higher relative abundance from small to larger size fractions with depth. For both, some ASVs were found to have a significantly higher relative abundance in PA (5–20 and >20 μm) fractions at depths below their FL (0.2–1.2 and 1.2–5 μm) maximum. This suggests that members of the FL community may have aggregated or been packaged into larger particles. *Cyanobacteria* have been found to contribute to carbon export in a manner proportional to their net primary production despite their small size [114], and more recent metabarcoding of particle microbial communities has revealed sequences of *Synechococcus* to be associated with particulate carbon export [87, 115, 116]. Lomas *et al*. [117] observed increased export of picophytoplankton at BATS, possibly due to increases in effective size and sinking rate. Unbalanced carbon fixation in the absence of sufficient inorganic nutrients can lead to increased production of exopolymeric substances by phytoplankton, including picophytoplankton [118]. Exopolymeric substances such as TEP can accentuate particle aggregation rates [107, 119–121], thereby increasing particle size and the potential sinking rate. These aggregates may also serve as a food source for zooplankton grazers, which repackage this material as fecal pellets, a process that contributes substantially to export production [122].

## Conclusion

We combined 16S rRNA gene metabarcoding with isotopic characterization of size-fractionated marine particles to better understand spatial variation in PA microbial communities and particle-mediated transformations of organic matter over depth at the BATS site. We observed clear and consistent differences in the isotopic signatures of organic matter transformations and microbial communities between size fractions. Smaller organic particles were found to be more heavily influenced by microbial processes, while larger particles showed evidence of transfer through higher trophic levels via zooplankton feeding and repackaging. Additionally, there was clear variation in community structure on particles between depths and size fractions. PA communities changed as much in depth as FL communities, while remaining distinct in composition. These observations did not fit with previous conceptual models based on the expectation that sinking particles transport taxa relatively rapidly across depth. Instead, our observations are consistent with two potential concepts. First, non-sinking particles are a significant fraction of total particles retrieved by pumps and are occupied by taxa specialized for particles that occupy a stratified depth range. Alternatively, the rapid turnover of particle microbial communities as they sink could explain our observations. We also observed increased differentiation between FL and PA communities below the DCM, hypothesized to be caused by differential reworking of more recalcitrant organic matter between fractions. Our data suggests that organic matter of varying size ranges undergo differing compositional transformations across depth, represented by differing microbial communities and particle biogeochemistry. Additionally, our methodology captures bulk trends in both sinking and suspended particles, the latter of which is systematically undersampled using sediment traps. This provides a deeper understanding of broader particle dynamics within the oligotrophic ocean.

## Supplementary Material

Supplementary_MaterialV2_ycae090

Supplementary_table_1_ycae090

## Data Availability

DNA sequence data are available in the National Center for Biotechnology Information (NCBI) Sequence Read Archive (SRA) under PRJNA1066814. R scripts and analytical pipeline can be found at https://github.com/jacquicomstock.
