# Supplementary material for: Marine particle size-fractionation indicates organic matter is processed by differing microbial communities on depth-specific particles
Source: ISME Commun. 2024 Jul 12;4(1):ycae090. doi: 10.1093/ismeco/ycae090 (PMC11334337; doi:10.1093/ismeco/ycae090)
Supplement: Supplementary_MaterialV2_ycae090 [file supplementary_materialv2_ycae090.docx]

**Supplementary Material**

**Supplementary Figures**


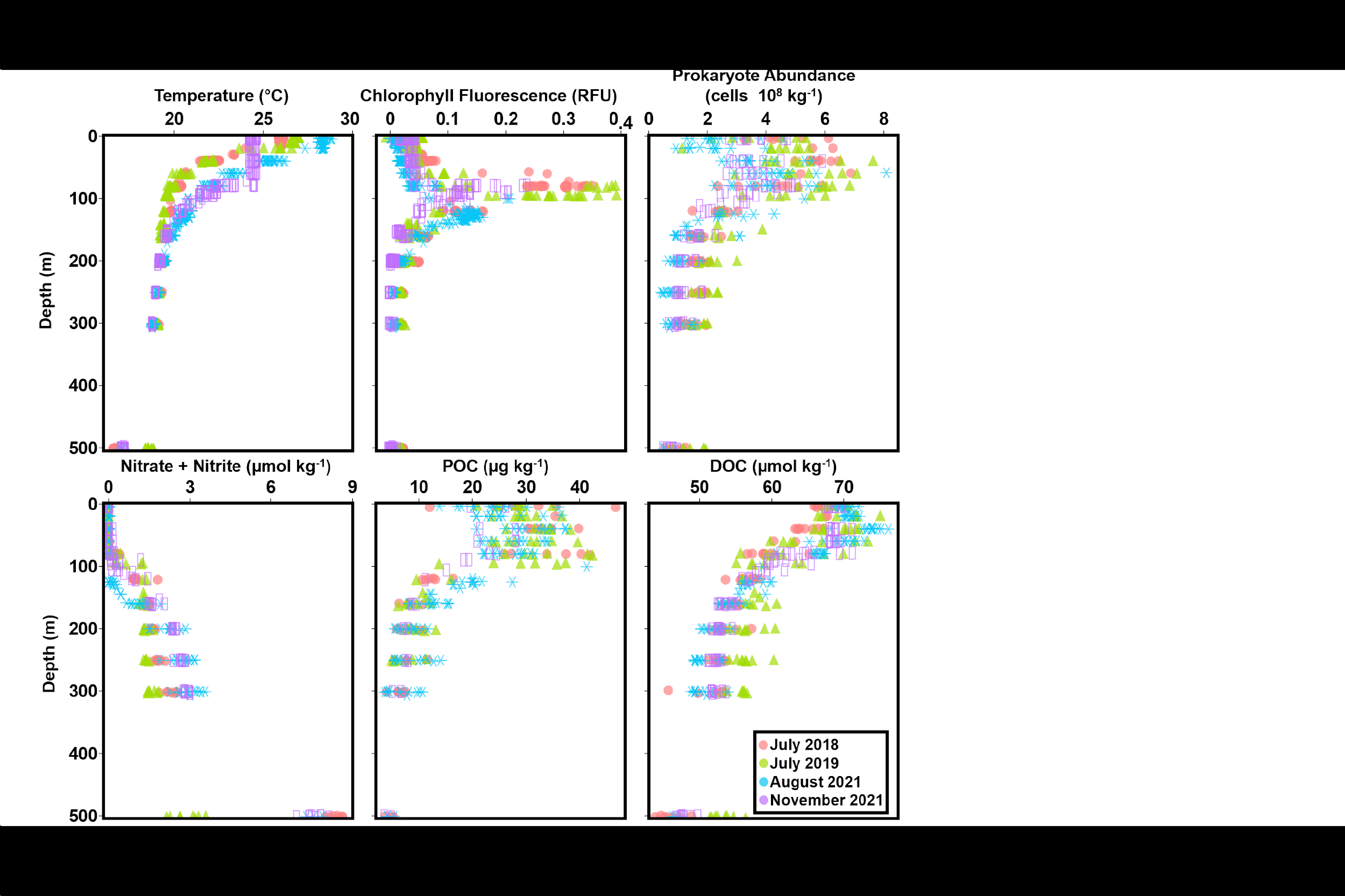


**Figure S1**: Depth profiles of the temperature, fluorescence, bacterial abundance, nitrate + nitrite, particulate organic carbon (POC) and dissolved organic carbon (DOC) across the upper 500 m of the water column during each of the time periods sampled.


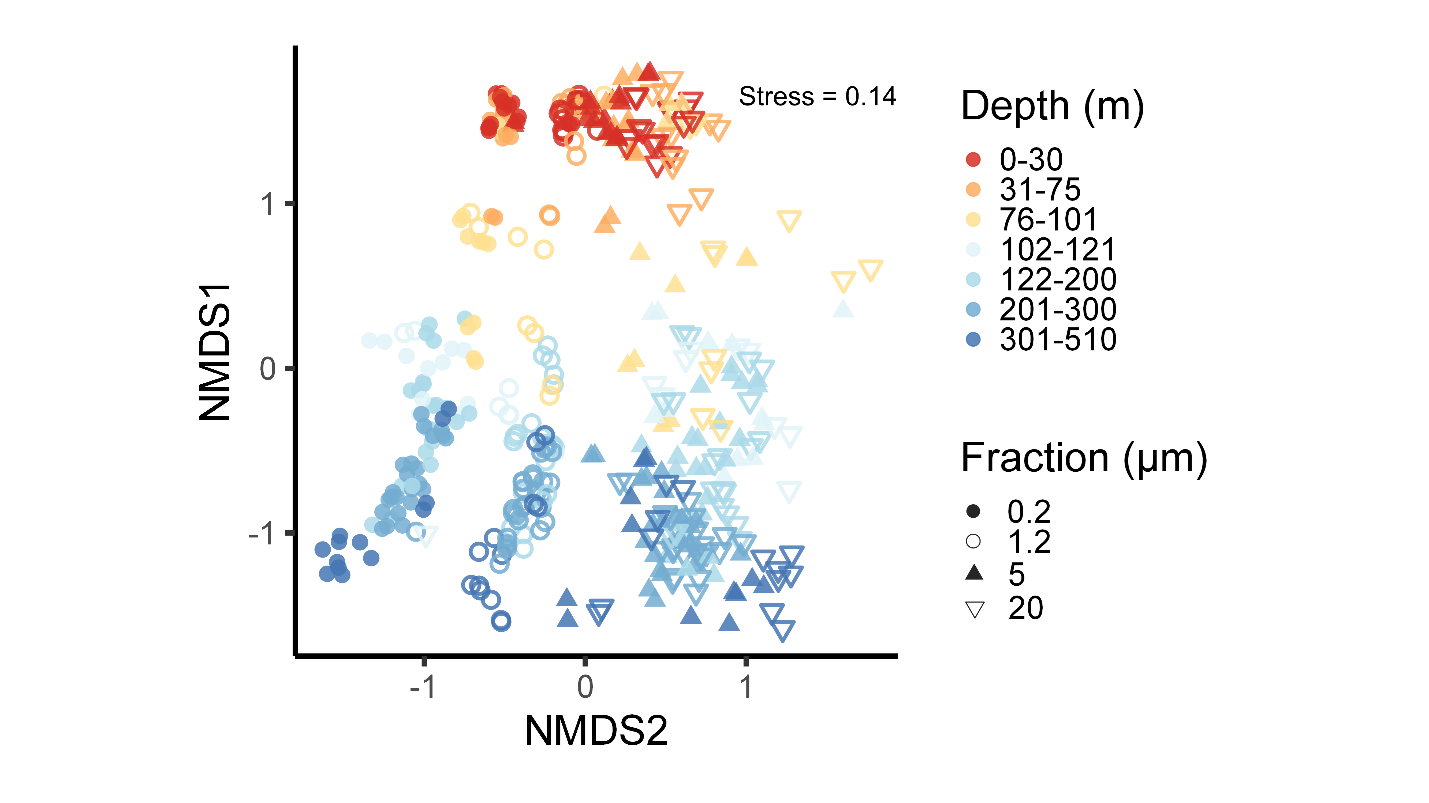


**Figure S2**: NMDS ordination of prokaryote communities based on 16S rRNA gene amplicons with cyanobacteria included


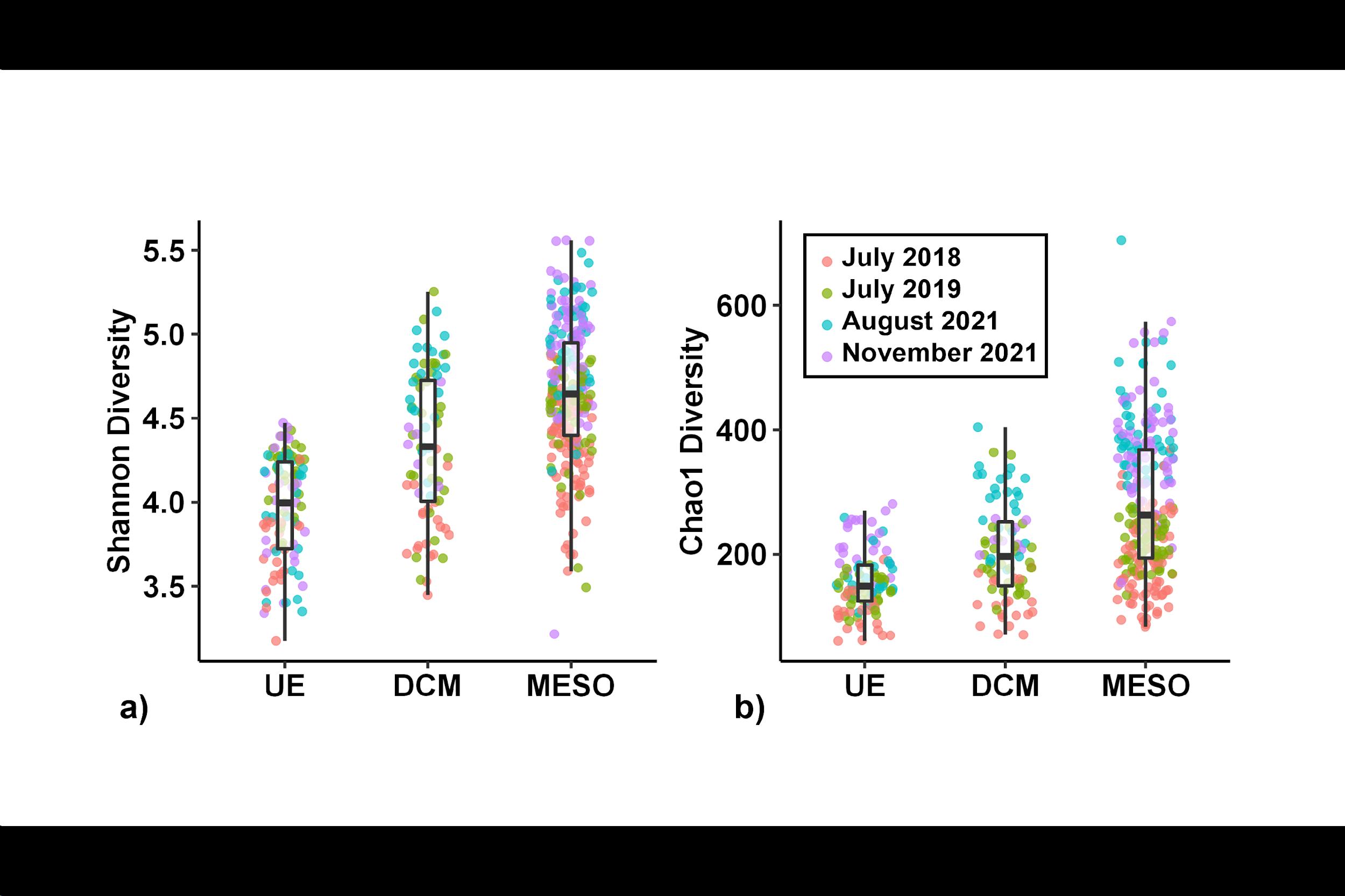


**Figure S3**: Alpha diversity of the 16S community of all size fractions with cyanobacteria


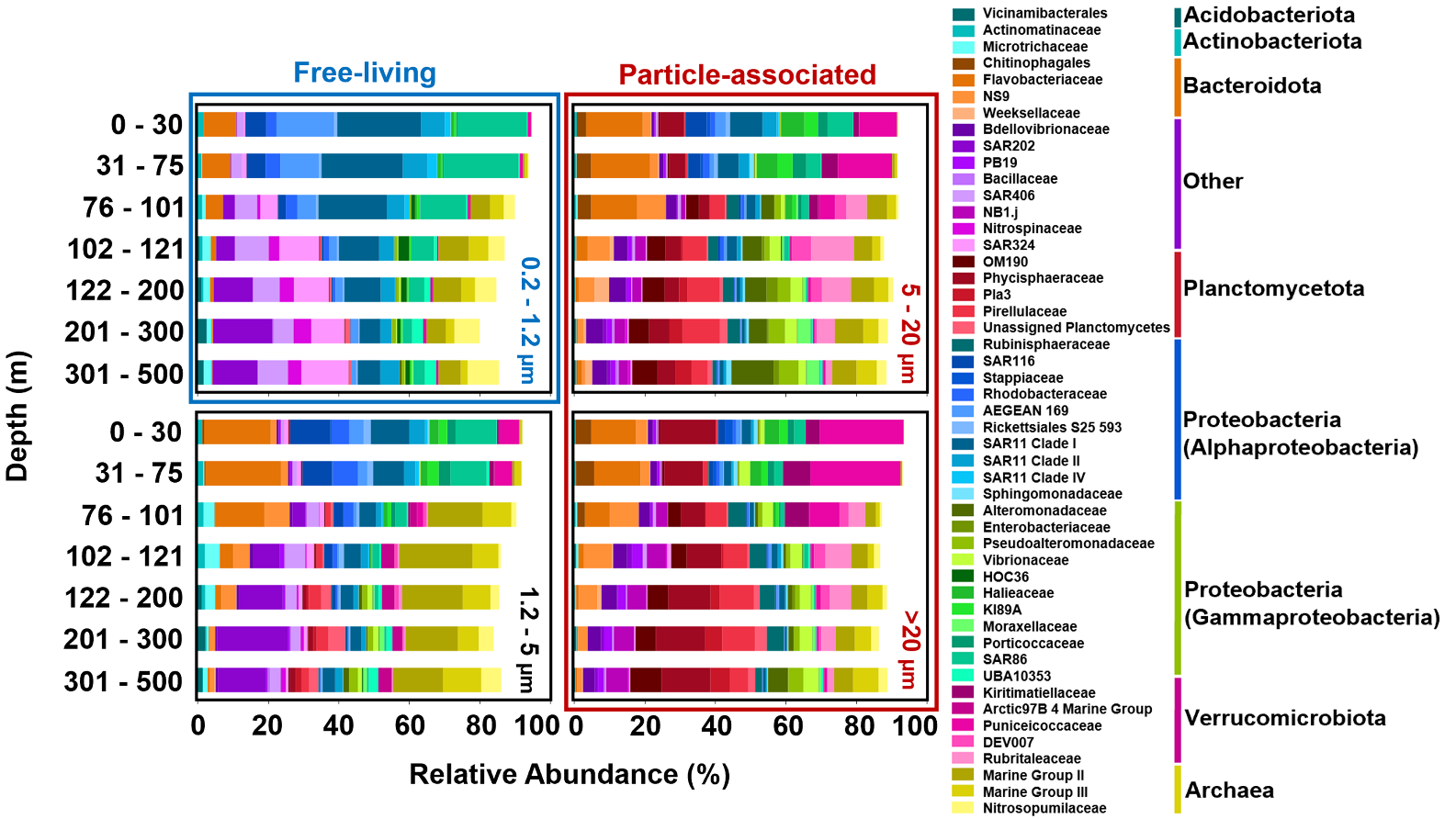


**Figure S4**: Relative abundances of microbial taxa (excluding cyanobacteria and plastids) grouped by family across size fractions and depth bins. Relative abundances are averages of depth bins taken across all four sampling times. Families that comprised less than 1% of community composition were excluded from visualization.


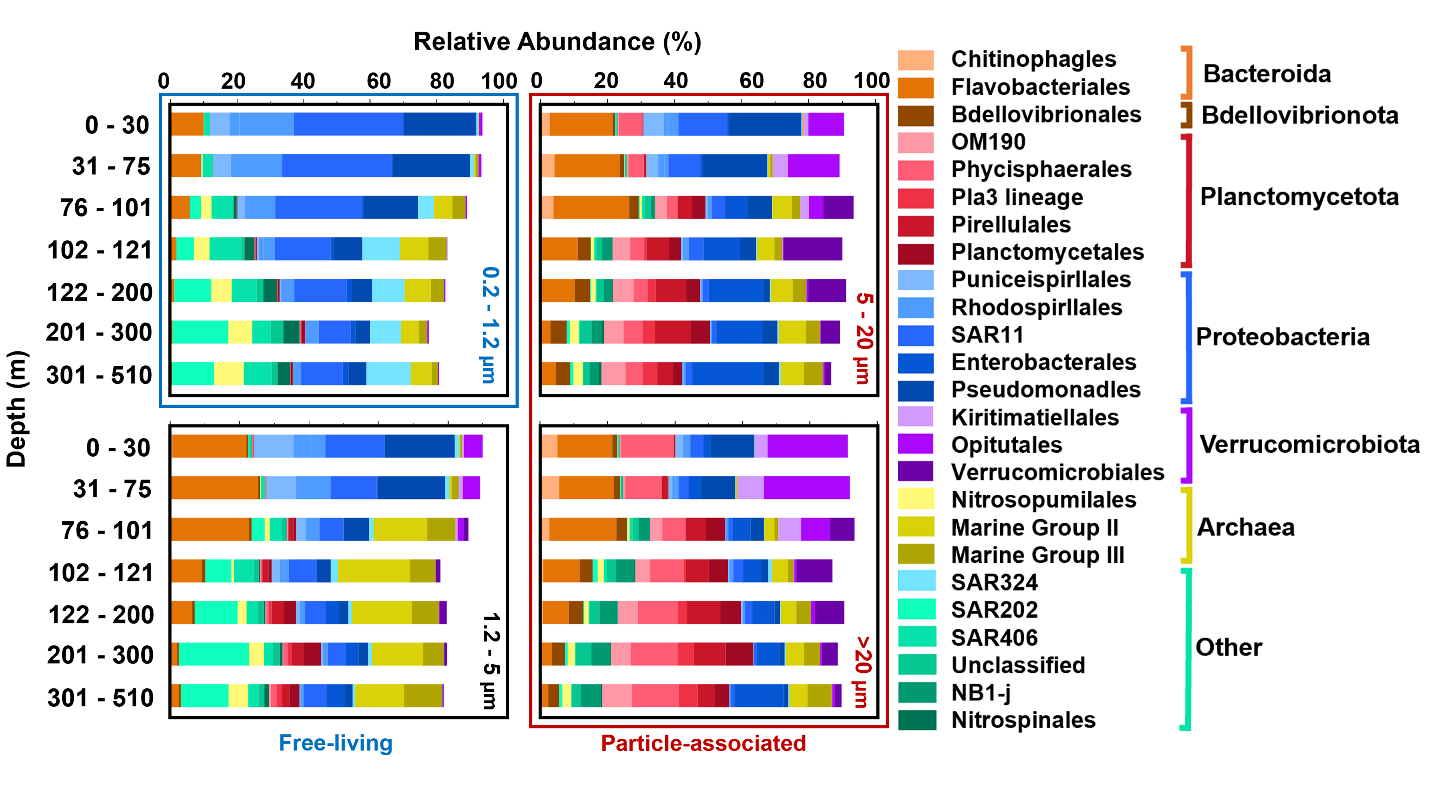


**Figure S5**: Average relative abundances of microbial taxa (excluding cyanobacteria and plastids) grouped by order across size fractions and depth bins. Orders that comprised less than 1% of community composition were excluded from visualization.


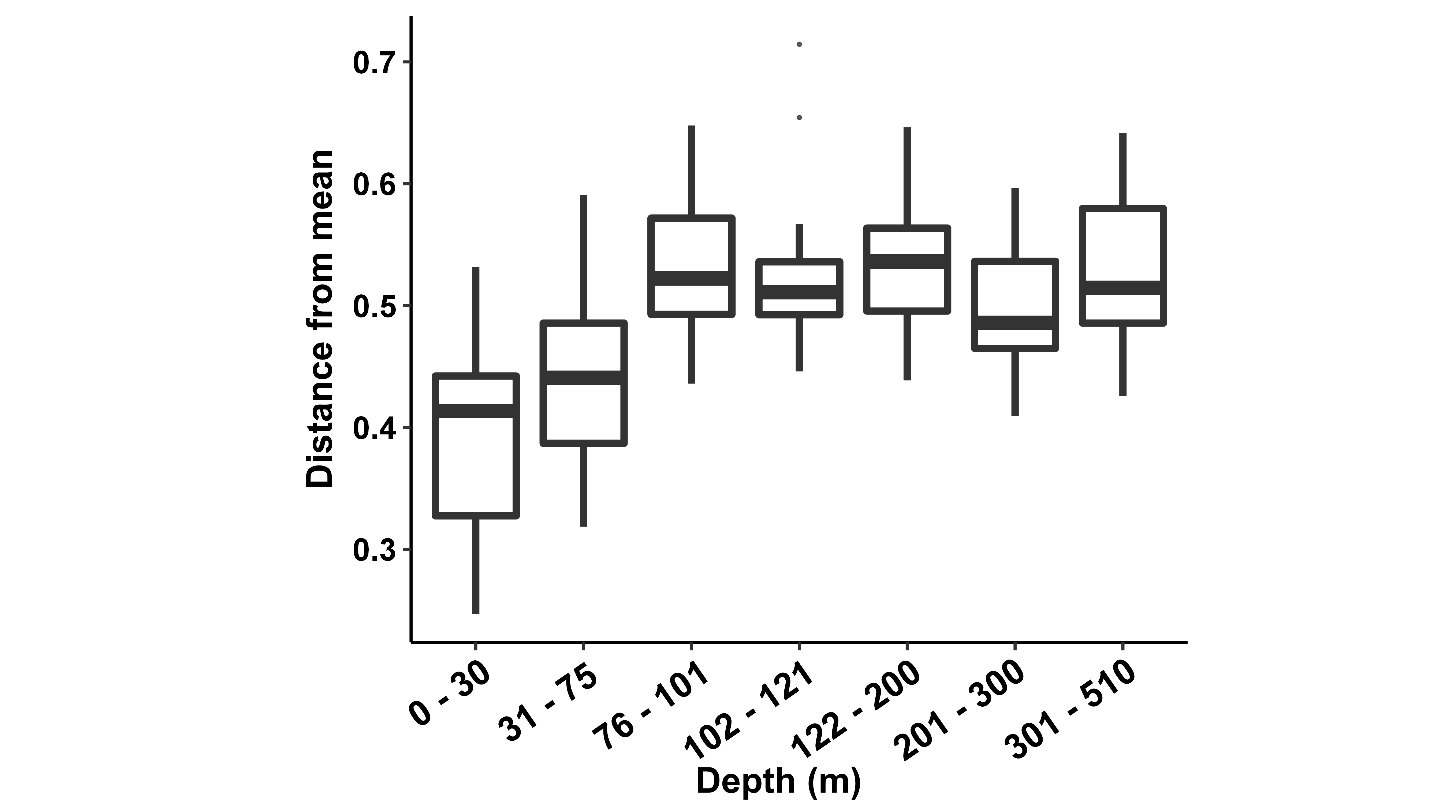


**Figure S6**: Distances from the mean community structure were calculated for each sample within each depth and values were visualized within depth bins. Communities within the upper water column (<75 m depth) displayed significantly (Tukey HSD p < 0.05) reduced distance to the mean than samples below 75 m.


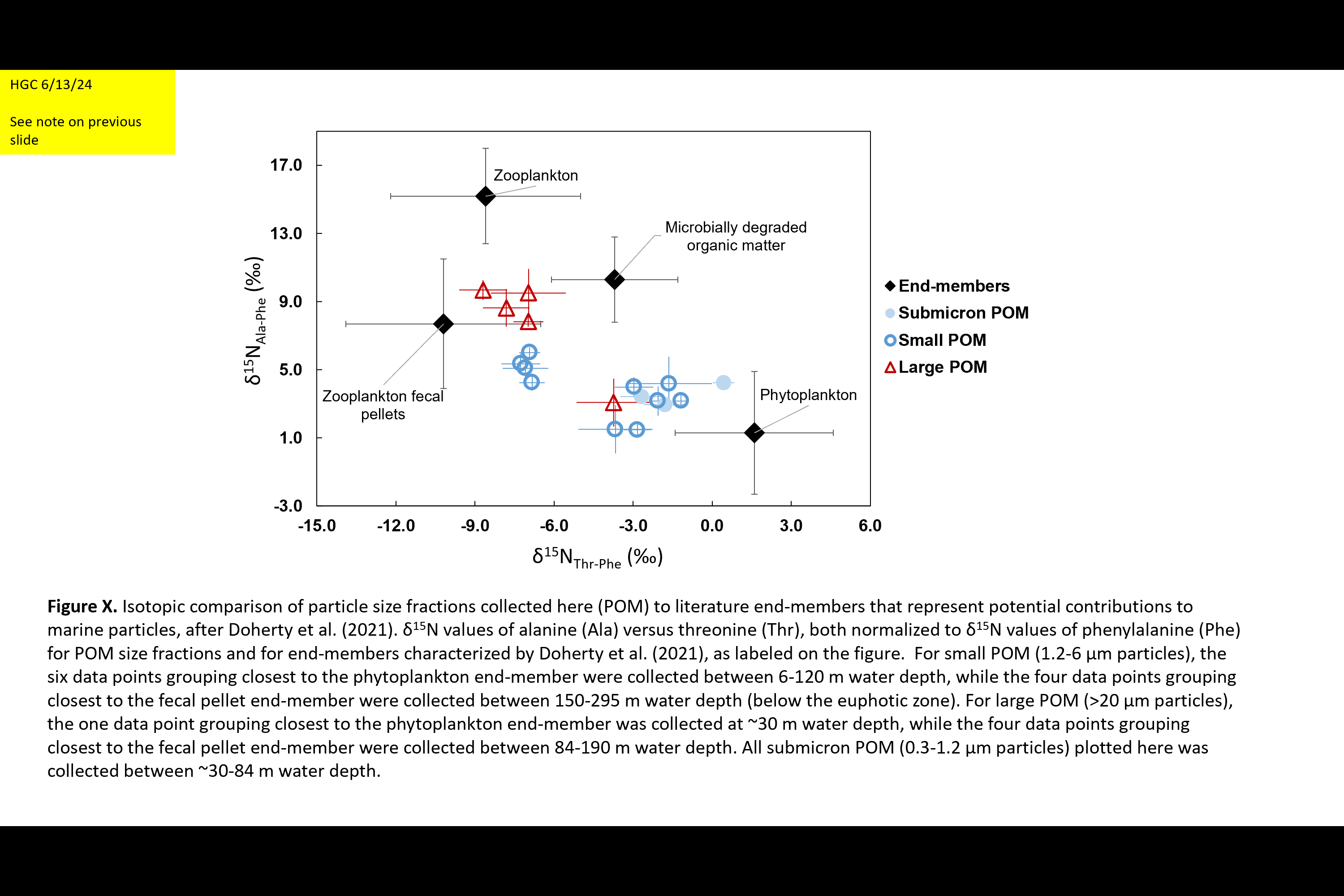


**Figure S7**: Isotopic comparison of particle size fractions collected here (POM) to literature end-members that represent potential contributions to marine particles, after Doherty et al. (2021). δ^15^N values of alanine (Ala) versus threonine (Thr), both normalized to δ^15^N values of phenylalanine (Phe) for POM size fractions and for end-members characterized by Doherty et al. (2021), as labeled on the figure. For small POM (1.2-6 μm particles), the six data points grouping closest to the phytoplankton end-member were collected between 6-120 m water depth, while the four data points grouping closest to the fecal pellet end-member were collected between 150-295 m water depth (below the euphotic zone). For large POM (>20 μm particles), the one data point grouping closest to the phytoplankton end-member was collected at ~30 m water depth, while the four data points grouping closest to the fecal pellet end-member were collected between 84-190 m water depth. All submicron POM (0.3-1.2 μm particles) plotted here was collected between ~30-84 m water depth.

**Supplementary Methods**

**Hydrographic measurements**

Water column hydrography was determined within hours before or after the in-situ pump deployments. The hydrographic data includes CTD sensor profiles of density, temperature, salinity, oxygen, and chlorophyll fluorescence along with discrete measurements of dissolved organic matter (DOM), particulate organic matter (POM), inorganic nutrients (NO_2_^-^ + NO_3_^-^; PO_4_^3-^), and bacterial abundance from Niskin bottles. The deep chlorophyll maximum (DCM) was defined as the depth (±10 m) with the highest chlorophyll fluorescence determined by the CTD Seabird fluorometer Chealsea Aqua 3 sensor.

**Inorganic nutrients** –

Unfiltered seawater samples were collected from 12 L Niskin bottles into 20 ml HDPE vials, frozen at -20˚C, and analyzed using flow injection analysis on a QuickChem 8000 (Lachat Instruments, Zellweger Analytics, Inc.) by the University of California, Santa Barbara Marine Science Institute Analytical Laboratory (detection limits: NO_2_^-^ + NO_3_^-^, 0.2 µmol L^-1^; PO_4_^3-^, 0.1 µmol L^-1^).

**Dissolved Organic Carbon**

DOC samples were collected, analyzed, referenced, and standardized according to Halewood et al., 2022. Briefly, seawater was filtered through precombusted (450°C) 47 mm GF/F filters and collected into precombusted 40 ml borosilicate glass vials. DOC samples were acidified with 4N HCl to pH ~3. DOC concentrations were analyzed, referenced, and standardized using high-temperature combustion on a modified TOC-V or TOC-L analyzer (Shimadzu) with precision of ~1 µmol L^-1^ or CV of 1-2%.

**Prokaryote Abundance**

Ten to forty ml of seawater was collected in sterile tubes (Falcon, fixed with 0.2µm filtered formalin (1% final concentration), and stored at -80°C until processing. Seawater was then filtered onto 0.2µm polycarbonate filters stained with Irgalan Black under ~100 mm Hg vacuum. Cells were stained with 5 µg ml^-1^ 4’,6’-diamidino-2-phenylindole dihydrochloride (DAPI) (Porter and Feig, 1980). Filters were mounted onto slides and enumerated under ultraviolet excitation using epifluorescence microscopy at 1000× magnification.
